# Rheumatological complaints in H syndrome: from inflammatory profiling to target treatment in a case study

**DOI:** 10.1186/s12969-023-00950-4

**Published:** 2024-01-23

**Authors:** Alessandra Tesser, Erica Valencic, Valentina Boz, Gianluca Tornese, Serena Pastore, Manuela Zanatta, Alberto Tommasini

**Affiliations:** 1 Department of Pediatrics, Institute for Maternal and Child Health, IRCCS Burlo Garofolo, via dell’Istria 65/1, Trieste, 34137 Italy; 2 Department of Medicine, Surgery and Health Sciences, University of Trieste, Strada di Fiume, 447, Trieste, 34149 Italy; 3 Centro di Coordinamento Regionale Malattie Rare ASUFC, Piazzale Santa Maria della Misericordia, Udine, 33100 Italy

**Keywords:** SLC29A3, H syndrome, Interferon, Arthritis, JAK inhibitors, Hydroxychloroquine

## Abstract

**Background:**

H Syndrome is a rare genetic condition caused by biallelic pathogenic variants in the SLC29A3 gene. It is characterized by a wide range of clinical manifestations, many of which are related to the immune-rheumatological field. These include scleroderma-like skin changes, deforming arthritis, and enlarged lymph nodes. The condition also features cardiac and endocrine defects, as well as hearing loss, for which the immune pathogenesis appears less clear. Immunomodulatory medications have been shown to improve many symptoms in recent experiences.

**Case presentation:**

A 21-year-old girl was referred to our institute after being diagnosed with H syndrome. Her medical history was characterized by the development of finger and toe deformities, which developed since the first years of life and progressively worsened with clinodactyly. At 6 years of age, she was diagnosed with diabetes mellitus without typical autoantibodies and with bilateral sensorineural hearing loss. She also complained of frequent episodes of lymphadenopathy, sometimes with colliquation and growth retardation due to pancreatic insufficiency. It wasn’t until the genetic diagnosis of H syndrome that the continual increase in acute phase reactants was noticed, suggesting that an immunological pathogenesis may be the source of her problems. During her visit to our institute, she reported serious pain in both feet and hands and difficulty walking due to knee arthritis and muscle contractures. Conventional therapy with steroid injection in affected joints and methotrexate only led to partial improvement. After a thorough assessment of her inflammatory profile showing a high interferon score, the girl received treatment with baricitinib. Furthermore, based on recent data showing that SLC29A3 deficiency results in interferon production because of Toll-like Receptor 7 activation in lysosomes, hydroxychloroquine was also added. The combination of the two drugs resulted for the first time in a rapid and persistent normalization of inflammatory markers, paralleled by a dramatic improvement in symptoms.

**Conclusions:**

We describe the results of inhibiting IFN inflammation in H syndrome and discuss how JAK inhibitors and antimalarials might represent a mechanistically based treatment for this orphan drug disorder.

## Background

H Syndrome, also known as Histiocytosis-Lymphadenopathy Plus Syndrome, is part of a range of recessive clinically related disorders due to biallelic pathogenic variants of the Solute Carrier Family 29 Member 3 (*SLC29A3*) gene (OMIM # 602,782) [1, 2]. The disease features cutaneous, cardiac, endocrine defects, joint contractures, deafness, and immune anomalies with huge phenotypic intrafamilial variability, witnessing the likely influence of multigenic and environmental factors in the development of symptoms [3]. Patients may experience pigmented hypertrichosis often with scleroderma-like changes, deforming arthritis with contractures, insulin-dependent diabetes mellitus, sensorineural deafness, lymphoproliferation, histiocytosis, and auto-inflammation [4]. According to recent reports, mycophenolate mofetil or tocilizumab medication may affect the course of the illness, leading to improvements in scleroderma-like skin lesions, arthritis, sensorineural deafness, and growth. The fact that these symptoms are affected by immunomodulatory medications is in line with their immune pathogenesis [5–7]. Anti-inflammatory medications, however, are frequently initiated many years after the onset of symptoms due to poor awareness of their rheumatological pertinence, making it challenging to assess their true efficacy in preventing illness-related organ damage. We aim to describe how inflammatory profiling may guide mechanistic driven treatment in a case of H syndrome.

## Case presentation

**Fig. 1:**
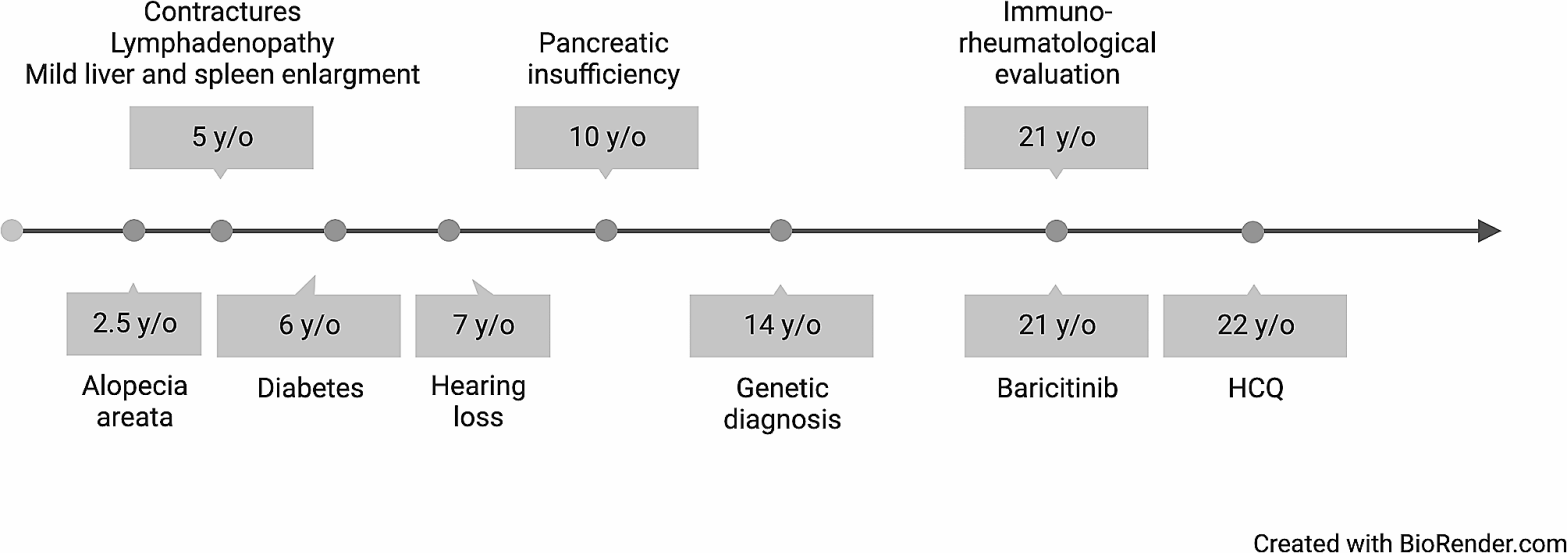
Timeline of clinical history and treatments (y/o: years old; HCQ; hydroxychloroquine)

A 21-year-old girl was referred to our Institute after a genetic diagnosis of H syndrome, due to compound heterozygosity for two pathogenic variants in *SLC29A3* gene (c.59_60dupCA,p.Ser21Glnfs*81, inherited from the father, not reported in online databases but resulting in destructed protein structure because of early frameshift and truncation; c.1087 C > T resulting in p.Arg363Trp, inherited from the mother, already reported as associated with H syndrome). She was born after an uneventful pregnancy from healthy unrelated parents. Her medical history (Fig. 1) was characterized by the development of deformation in the fingers and toes, progressively worsening with clinodactyly, for which she underwent flexor lengthening surgery (last three fingers right hand) at 5 years of age, without clear benefit.

There was no clear clinical sign of arthritis, and the picture was interpreted as arthrogryposis, for which she underwent calcaneal filing. A malformation syndrome was hypothesized. Considering the presence of a mild liver and spleen enlargement, she underwent several examinations, including Nuclear Resonance imaging of abdomen and brain and metabolic analyses to rule out lysosomal storage diseases. She also complained of frequent episodes of lymphadenopathy, sometimes with colliquation, without a clear history of recurrent infections. At 6 years of age, she was diagnosed with diabetes mellitus, without typical autoantibodies. In the following months, progressive bilateral sensorineural hearing loss was also noticed, leading some years later to the use of hearing aids. At 10 years, a slowdown in growth led to the detection of pancreatic insufficiency requiring pancreatic enzyme supplementation. Unfortunately, no rheumatological evaluation was requested before the genetic diagnosis of H syndrome.

**Fig. 2:**
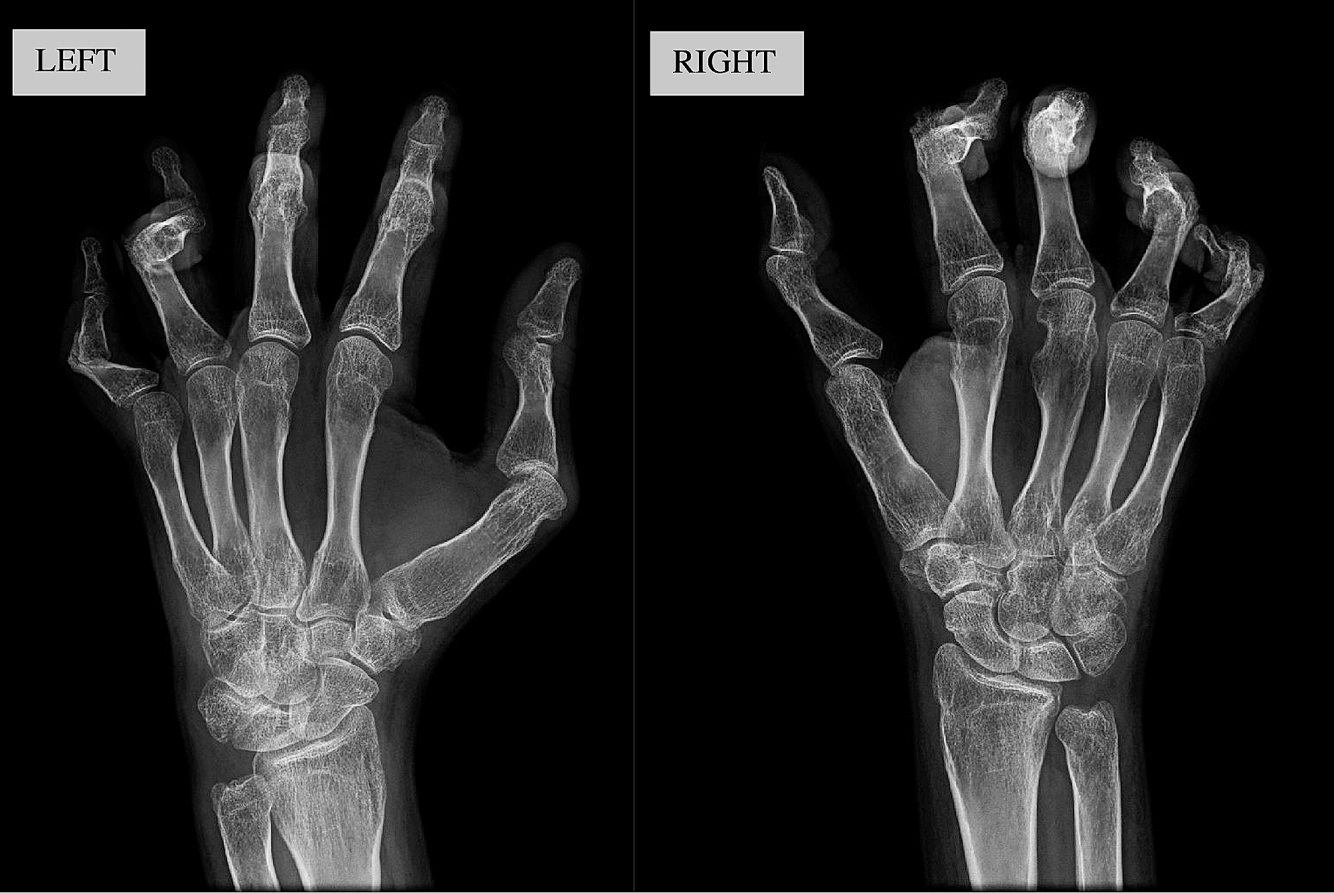
Hands X-rays showed deforming arthropathy with multiple subluxations but without significant erosion

At the visit at our clinics, the girl reported difficulty walking, with the inability to walk for more than 5 min due both to knee arthritis and muscle contractures. At physical examination it was noticeable a mild limitation of hips intrarotation. She complained of serious pain at both feet and hands and X-rays showed a deforming arthropathy (Fig. 2).

Considering the genetic diagnosis that the girl received 7 years before our visit, together with recent data on the immune pathogenesis of the disease and the potential of immunomodulation, we performed a thorough assessment of immunity and inflammation. The blood examinations showed a mild elevation of Erythrocyte Sedimentation Rate (ESR, 47 mm/h, normal value < 20) and C-Reactive Protein (CRP, 15.6 mg/L, normal value < 5 mg/mL) with normal levels of immunoglobulins (IgG 1433 mg/dL, IgA 216 mg/dL, IgM 40 mg/dL) and negative results for common autoantibodies. Interferon (IFN) Score (IS) was calculated by real time PCR as the median fold expression of the following interferon stimulated genes (*IFI27*, *IFI44L*, *IFIT1*, *ISG15*, *RSAD2*, and *SIGLEC1*) compared to a pool of controls [8]. The finding of a high IS, (23.7, normal value < 2.2) was consistent with IFN-mediated inflammation. Immunophenotyping revealed near-normal levels of T cells (CD4 442 cells/µL; CD8 412 cells/µL, recent thymic emigrants CD45RA + CD31 + 101 cells /µL) with a defect in memory B cells (CD19 + CD27 + cells 3.81% of CD19 + cells; normal values 4–55%) and mildly elevated percentages of double negative alpha/beta T cells (3.64% of CD3 + cells, normal values < 3%).

Antinuclear antibodies, extractable nuclear antigen antibodies, islet cell and thyroid related autoantibodies were all negative. Based on the presence of deforming active arthritis with IFN-mediated inflammation, we injected both knees with triamcinolone hexacetonide and started an antirheumatic treatment with baricitinib (4 mg once in day) and subcutaneous methotrexate (15 mg/week = 10 mg/m2). Even if tocilizumab had reported somehow effective in H syndrome, we chose to administer a JAK inhibitor to contrast IFN in addition to IL-6 related inflammation. Despite some clinical improvement, after 6 months the patient still complained of diffuse arthralgias and muscular pain with difficulty walking, which were paralleled by persistently elevated inflammatory markers (ESR 73 mm/h, CRP 39.6 mg/L, IS 22.3). We wondered if it could be better increasing the dosing of baricitinib on a twice in day regimen or adding a second medication active on the diseased pathway. We thus reviewed recent studies, showing that the defective transport of nucleosides across the membrane of SLC29A3-deficient lysosomes can lead to increased Toll-Like Receptor 7 (TLR7) activation and IFN inflammation [9–10]. We thus speculated that hydroxychloroquine (HCQ), a conventional treatment in lupus-spectrum arthritis, might contrast the disease-associated inflammation by accumulating in lysosomes and inhibiting TLR7 activation. Five months after the switch from methotrexate to HCQ (400 mg/day = 6.5 mg/Kg), the girl reported a dramatic improvement in her symptoms, with substantial improvement in walking ability and normalization of all inflammatory indexes (CRP decreased to 1.8 mg/L, ESR to 15 mm/h and the IS dropped to 1.4). Physical examination of hips showed a complete range of motion. Immunophenotyping showed a trend increase in memory B cells (CD19 + CD27 + 6% of CD19 + cells). One year after the start of the antirheumatic treatment the girl appears in good condition, with reduced pain and improved functional capacity.

## Discussion and conclusions

We described the immune profile of a girl with H syndrome and discussed how it influenced therapeutic choices. Personalized, mechanistically based, treatment can be of valuable utility in subjects with rare disorders for which poor therapeutic options are available. In our case, similarly to what is reported in the literature, the disease is accompanied by a constant alteration in acute phase reactants and by various minor abnormalities in lymphocyte subsets. Compared with previous reports, we showed a mild deficiency in memory B cells and a slight elevation of Double Negative T cells (DNTs) [11]. DNTs have not been assessed so far in H syndrome, but their increase may be coherent with the disease-associated lymphoproliferation and with perturbed mTOR homeostasis observed in cellular models of SLC29A3 deficiency [12]. Although transcriptomic studies in patients with H syndrome highlighted an IFN signature in peripheral blood cells, the Interferon Score (IS) has not been previously calculated and exploited for therapeutic stratification in patients [13]. We showed that the inflammatory response in our patient was associated with a high IS and that it could be favorably influenced by targeted treatment with Janus Kinase (JAK) inhibitor and hydroxychloroquine, which also led to overall clinical benefit.

Even if this is a single case report, it might have great importance for other people with this rare and neglected disease. Indeed, the disease may develop slowly, and its inflammatory nature may run unnoticed for a long time, leading to irreversible organ damage. High levels of Interleukin 6 (IL6) have been previously described to be associated with H syndrome, and treatments targeted to this cytokine have been proven effective in contrasting various disease-associated features. However, treatments have not been focused so far on IFNs, which can also be crucial drivers in pathogenesis. Indeed, the scarce availability of routine assays to measure the IS might hinder a timely recognition of this type of inflammation, delaying proper treatment.

We speculated that JAK inhibitors, being active both on IFNs and on IL6, could have a stronger potential as a disease modifier in H syndrome compared with tocilizumab. However, in our case, the addition of baricitinib led only to a slight improvement in the rheumatological complaints reported by the girl, which might be the result of several years of untreated inflammation. Indeed, IFN-associated arthropathies often show the characteristics of deforming non-erosive arthritis that can develop slowly in the years leading to irreversible contractures like in Systemic Lupus Erythematosus (SLE)-associated Jaccoud arthropathy [14, 15]. Moreover, scleroderma-like skin changes can also contribute to joint stiffness in H syndrome [16]. Even if JAK inhibitors might be a reasonable option to contrast the progression of the disease, once the disease has evolved to contractures and deformities, there is scarce room for therapies. This is the reason why it is so important to timely detect the inflammatory nature of the osteoarticular complaints described in H syndrome.

Interestingly, treatment with baricitinib in our case did not lead to any improvement of inflammatory markers, including IS. Conversely, the addition of HCQ was paralleled by a stable normalization of ESR, CRP, and IS, which were maintained at one year of follow-up, together with a subjective improvement in the patient’s reported symptoms. Although we describe an isolated observation made on a single patient, we believe that it is worthy of discussion considering recent insights from in vitro studies on H syndrome.

The product of the *SLC29A3* gene is ENT3, which is involved in the transport of nucleosides across lysosomal and mitochondrial cell membranes. Mutations involved in H syndrome are associated with the loss of transport of adenosine, derived from autophagic or phagocytic pathways, through lysosomal membranes. This is associated with spontaneous and progressive macrophage-dominated histiocytosis in mice with paradoxical activation of macrophages in response to apoptotic signals [17]. Moreover, the deletion of *SLC29A3* in preclinical studies is associated with impaired autophagic regulation and altered proliferative potential in stem cells, which may account for some of the syndromic features described in H syndrome [12]. In addition, according to recent research, the defective function of ENT3, leading to accumulation of adenosine in lysosomes, could lead to abnormal stimulation of TLR7 and TLR8, resulting in MAPK signaling and activation of an inflammatory response driven by IFNs [9, 10, 18]. Of note, HCQ is a weak base that accumulates in the lysosomes of immune cells, where it exerts its action by interacting with membrane stability, inhibiting the proteolytic processing of TLR7 and thereby its activation [19–21].In this, HCQ may target quite directly the underlying molecular pathway involved in inflammation in H syndrome, providing hope for individuals suffering from this rare disorder.

It is not surprising that HCQ is also a consolidated treatment in SLE where it is believed to act by reducing the lysosomal stimulation of Toll-like receptors and the consequent inflammatory response. Interestingly, recent data suggested that defects in *SLC29A3* may contribute to the pathogenesis of SLE by affecting lysosome function in monocytic cells [22], thus providing a possible shared rationale for the use of antimalarials in SLE and H syndrome. Even if the use of HCQ has been reported in 2 cases of H syndrome, its use was not associated with JAK inhibitors and there is no detail on the biological and clinical response in these cases [23–24].

Recent data suggest that an alternative strategy may rely in targeting MAPK with MEK inhibitor therapy, which led to resolution of histiocytosis and inflammation in a patient with H syndrome [18].

H syndrome is a rare disorder and patients may be referred to various specialists, including orthopedists, hematologists, and pediatricians experienced on rare complex syndromes. However, given the role of immune pathogenesis, the importance of a proper assessment of the inflammatory profile, and the potential of immunomodulatory therapies, it is crucial that patients are referred to immuno-rheumatologists as soon as possible. Indeed, most organ damage develops progressively on the background of an immune pathogenesis. As discussed, articular manifestations are reminiscent of those found in monogenic interferonopathies, in SLE, or systemic sclerosis, and can at least in part be prevented by appropriate treatments. Assessment of interferon-mediated inflammation can be performed by calculating the interferon score by real time PCR analysis. Of note, other interferon-related markers can also by analyzed, e.g., the measure of Siglec-1 expression by flow cytometry or the expression of chemokines like CXCL9 and CXL10 by ELISA [25, 26].

Even if we have no definite proof of this, it is logical to assume that progressive deafness and endocrine features can also recognize immune pathogenesis. Deafness is reported to occur from early childhood in 53% of patients with H syndrome and its immune pathogenesis is witnessed by the improvement of some cases after tocilizumab treatment [27]. It is not possible to forecast if targeting also IFNs may result in a better improvement of hearing loss, but it is worth noting that JAK inhibitors can target IL6 together with IFNs, proposing themselves as good candidates also for this aspect. Furthermore, it was shown that IFN gamma can sensitize cochlear cells to inflammatory damage [28].

Insulin-dependent diabetes, usually not associated with autoantibodies, is also common in H syndrome and might also recognize inflammatory pathogenesis, possibly driven by IFNs. Indeed, diabetes can develop in the absence of typical autoantibodies in subjects with various interferonopathies, including DNase2 deficiency and Signal Transducer And Activator Of Transcription 1 (STAT1) gain-of-function (GOF) immunodeficiency [29–31]. Moreover, IFN induced with helicase C domain 1 (*IFIH1*), which is an interferonopathy-related gene, has been involved in accelerating the development of diabetes after viral infections [32]. Interestingly, JAK inhibitors can reverse the development of diabetes in subjects with STAT1 GOF, even if their success might depend on overall immunosuppression and not just on the inhibition of IFN signaling [33]. We cannot be certain of the responsibility of IFNs in the pathogenesis of diabetes in H syndrome, but the hypothesis is very attractive and maybe a further reason to justify a trial with JAK inhibitors and hydroxychloroquine in an early stage of the disease. We believe that block TLR7 signaling using HCQ in H syndrome need further exploration in a much larger cohort of patients. In conclusion, we highly recommend performing a thorough inflammatory profiling in patients with H syndrome as early as possible and considering treatments like those based on tocilizumab, JAK inhibitors, and HCQ that might prevent the development of disease-associated organ damage.

## Data Availability

Data is available from the corresponding author upon reasonable request.

## Abbreviations

CRP: C-reactive protein
DNTs: Double Negative Cells
ENT3: Equilibrative nucleoside transporter 3
ESR: Erythrocyte sedimentation rate
IL6: Interleukin 6
IFN: Interferon
IS: Interferon score
JAK: Janus kinase
SLC29A3: Solute carrier family 29 member 3
SLE: Systemic lupus erythematosus
STAT1: Signal transducer and activator of transcription 1
TLR7: Toll-like receptor 7
